# Supplementary material for: Adjuvant Chemoradiotherapy or Radiotherapy Alone for Early Squamous Cervical Cancer with a Single Surgical-Pathological High-Risk Factor
Source: Cancers (Basel). 2025 Jun 18;17(12):2041. doi: 10.3390/cancers17122041 (PMC12190927; doi:10.3390/cancers17122041)
Supplement: Supplementary file 1 [file cancers-17-02041-s001.zip › cancers-3616375-supplementary.pdf]

Supplementary

Figure S1. Standardized difference of propensity score model covariates before and after adjustment.

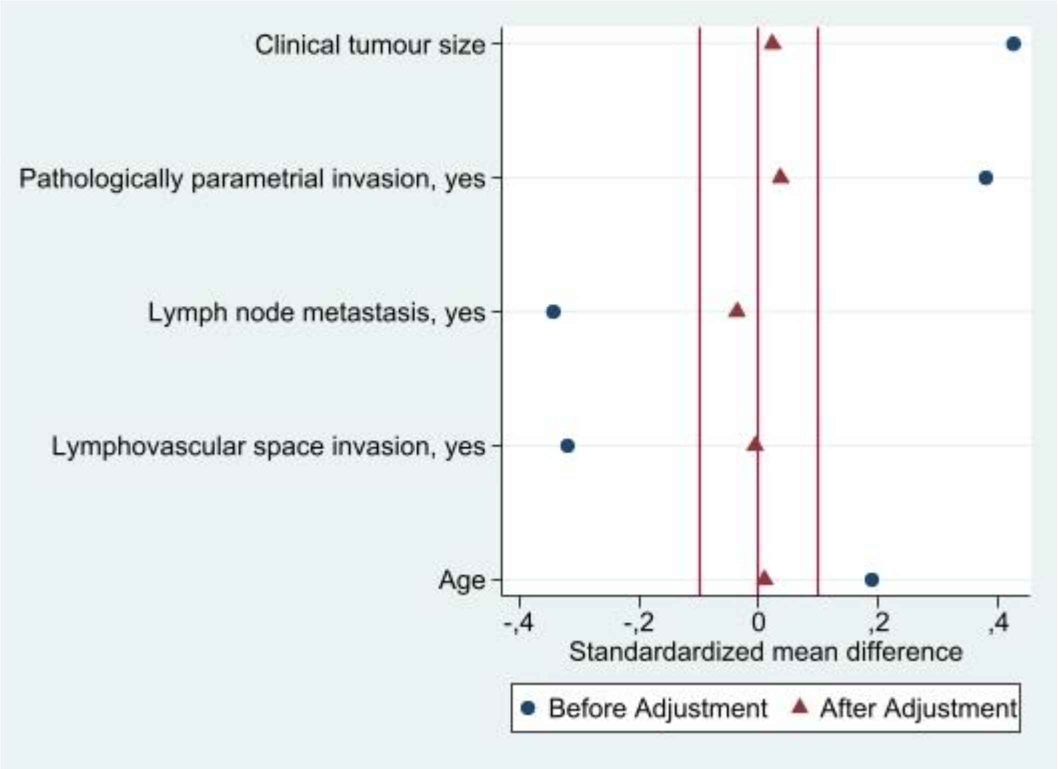

Table S1. Cox regression survival sensitivity analyses.

Analysis type		Recurrence-free survival		
		HR	95% CI	p-value
IPTW for locoregional recurrences	CRT	1.00	Reference	
	RT	0.09	0.01-0.79	0.03
IPTW for distant recurrences	CRT	1.00	Reference	
	RT	0.85	0.21-3.37	0.81
IPTW for open surgery	CRT	1.00	Reference	
	RT	0.58	0.18-1.87	p=0.37

Univariate for surgical approach	Open	1.00	Reference	
	Minimally invasive	0.74	0.29-1.87	0.52

		Overall survival		
IPTW for open surgery	CRT	1.00	Reference	
	RT	0.55	0.19-1.63	0.28
Univariate for surgical approach	Open	1.00	Reference	
	Minimally invasive	0.74	0.29-1.87	0.52

Abbreviations: IPTW, inverse probability treatment weighting; RT, radiotherapy; CRT chemoradiotherapy; HR, hazard ratio; CI, confidence interval.
